# Early disease progression of Hurler syndrome

**DOI:** 10.1186/s13023-017-0583-7

**Published:** 2017-02-14

**Authors:** Bridget T. Kiely, Jennifer L. Kohler, Hannah Y. Coletti, Michele D. Poe, Maria L. Escolar

**Affiliations:** 1 Program for the Study of Neurodevelopment in Rare Disorders, Children’s Hospital of Pittsburgh of UPMC, 4401 Penn Ave, Pittsburgh, PA 15224 USA; 2 University of North Carolina School of Medicine, Chapel Hill, NC 27599 USA

**Keywords:** Mucopolysaccharidosis type I, MPS I, Hurler syndrome, lysosomal storage disorders, Newborn screening, Natural history

## Abstract

**Background:**

Newborn screening for mucopolysaccharidosis type I (MPS I) shows promise to improve outcomes by facilitating early diagnosis and treatment. However, diagnostic tests for MPS I are of limited value in predicting whether a child will develop severe central nervous system disease associated with Hurler syndrome, or minimal or no central nervous system involvement associated with the attenuated phenotypes (Hurler–Scheie and Scheie syndromes). Given that the optimal treatment differs between Hurler syndrome and the attenuated MPS I phenotypes, the absence of a reliable prognostic biomarker complicates clinical decision making for infants diagnosed through newborn screening. Information about the natural history of Hurler syndrome may aid in the management of affected infants, contribute to treatment decisions, and facilitate evaluation of treatment effectiveness and prognosis. Thus, the aim of this study was to characterize the progression and timing of symptom onset in infants with Hurler syndrome.

**Results:**

Clinical data from 55 patients evaluated at a single center were retrospectively reviewed. Information about each child’s medical history was obtained following a standardized protocol including a thorough parent interview and the review of previous medical records. All patients underwent systematic physical and neurodevelopmental evaluations by a multidisciplinary team. Nearly all patients (98%) showed signs of disease during the first 6 months of life. Common early disease manifestations included failed newborn hearing screen, respiratory symptoms, difficulty latching, and otitis media. Other symptoms such as kyphosis, corneal clouding, cardiac disease, joint restrictions, and enlarged head circumference typically appeared slightly later (median age, 8–10 months). During the first 12 months, gross motor development was the most severely affected area of functioning, and a significant number of patients also experienced language delays. Cognition was typically preserved during this period.

**Conclusions:**

In this large cohort of patients with Hurler syndrome, the vast majority showed signs and symptoms of disease during the first months of life. More research is needed to determine the extent to which early clinical manifestations of MPS I can predict phenotype and treatment outcomes.

## Background

Mucopolysaccharidosis type I (MPS I, OMIM 252800) is a rare lysosomal storage disorder caused by the deficiency or complete absence of enzyme α-L-iduronidase activity. Inadequate activity of this enzyme leads to the accumulation of glycosaminoglycans throughout the body, resulting in progressive multisystem deterioration. Although MPS I is associated with a continuum of disease presentations, it has traditionally been divided into three clinical phenotypes. Hurler syndrome, the most severe form, typically manifests during the first year of life. Affected children rapidly develop significant cognitive impairment and somatic disease in multiple organ systems, leading to death within the first decade in the absence of treatment. The attenuated forms of MPS I, known as Hurler–Scheie syndrome and Scheie syndrome, are characterized by later onset of symptoms, longer life expectancy, and mild or no central nervous system (CNS) involvement. Symptoms found across the entire MPS I spectrum include corneal clouding, organomegaly, cardiac valve abnormalities, joint contractures, and dysostosis multiplex [1].

Treatment for MPS I is based on clinical presentation. Intravenous enzyme replacement therapy (ERT) is the first-line treatment for individuals with the attenuated forms of this disorder [2]. In these patients ERT has been shown to improve quality of life and ameliorate hepatomegaly, apnea, joint restrictions, and other somatic symptoms [3–5]. However, because the recombinant enzyme cannot cross the blood–brain barrier, ERT alone is generally not sufficient to manage the CNS manifestations associated with Hurler syndrome. For these patients, hematopoietic stem cell transplantation (HSCT) is the mainstay of treatment, although ERT may be added as an adjunct therapy [2]. Poe et al. (2014) found that patients with Hurler syndrome who underwent HSCT prior to 9 months of age showed relatively normal trajectories of cognitive development, whereas those who were transplanted later experienced long-term deficits [6]. These findings suggest that HSCT may be able to halt the progression of cognitive decline but is less effective at reversing pre-existing neurological damage. Thus, early treatment of affected individuals is crucial.

Historically, most patients with MPS I were diagnosed only after the appearance of characteristic disease manifestations. However, pilot newborn screening (NBS) programs for this disorder have recently been implemented in a number of locations, including Taiwan and Italy [7–9]. Moreover, in February 2016 the U.S. Secretary of Health and Human Services approved MPS I for addition to the Recommended Uniform Screening Panel. This development is likely to result in increased implementation of newborn screening for this disorder in the coming years.

Early diagnosis of patients with MPS I shows promise to improve outcomes. Nonetheless, the implementation of routine newborn screening for this disorder will likely pose a number of challenges for clinicians. To select the most appropriate disease-modifying therapy (HSCT and/or ERT) for each patient, clinicians will need to determine which infants are most likely to develop the severe CNS involvement associated with Hurler syndrome. However, the limited prognostic value of genetic and biochemical tests complicates phenotype prediction at the time of diagnosis for many patients [10–12]. In the absence of a biomarker that can reliably predict disease course, patients diagnosed with MPS I through newborn screening must be monitored closely during the first months of life.

Although only a subset of MPS I patients develop the most severe phenotype, information about the natural history of Hurler syndrome is relevant to the clinical management of all newborns with MPS I. Knowledge of the early natural history of Hurler syndrome may help to limit delays between symptom onset and initiation of appropriate supportive treatments, and enable clinicians to proactively monitor all newborns with MPS I for common early disease manifestations. Characterization of the earliest manifestations of Hurler syndrome may also contribute to the identification of prognostic clinical markers. These markers, in combination with other test results, may help clinicians determine which infants are the best candidates for HSCT and which should be treated with less aggressive therapies such as ERT. More data on the progression of early signs and symptoms of Hurler syndrome are needed to serve as a comparator for the evaluation of treatment effectiveness and to assess the true clinical benefit of NBS once it is implemented. However, because of the rarity of Hurler syndrome, there is a paucity of research about its natural history [1, 12–14]. Thus, the aim of this study was to characterize the onset and progression of neurological and somatic disease in a large cohort of patients with Hurler syndrome who underwent standardized multidisciplinary evaluations at a single center.

## Methods

### Inclusion and exclusion criteria

Clinical data were retrospectively reviewed from all patients with a confirmed diagnosis of Hurler syndrome that were seen at the Program for the Study of Neurodevelopment in Rare Disorders between 2000 and 2013 (*n* = 55). Diagnosis was based on deficiency or complete absence of enzyme α-L-iduronidase activity, increased glycosaminoglycans in urine, and mutation analysis predictive of phenotype, Hurler phenotype or family history of Hurler syndrome. Only data collected before treatment with HSCT were included in the analysis.

### Data acquisition

Disease onset and clinical course were characterized by reviewing available outside medical records, parental responses to a detailed questionnaire, and clinical data collected at the time of evaluation at our specialized program. The parental questionnaire covered the child’s prenatal and neonatal history, acquisition of developmental milestones, behaviors, initial signs of disease, and prior diagnoses, surgeries, and hospitalizations. When available, the results of cardiac evaluations, brain and cervical spine magnetic resonance imaging (MRI) scans, and audiological assessments were also reviewed.

At each clinic visit, the patients underwent comprehensive physical and neurodevelopmental evaluations performed over the course of 4–6 h by a team of specialists including neurodevelopmental pediatricians, speech-language pathologists, physical and occupational therapists, psychologists, and audiologists. Standardized, validated tests that are optimal for use in children with lysosomal storage disorders were administered to assess function across several developmental domains (i.e., cognition, adaptive behavior, expressive and receptive language, and gross and fine motor skills) [15]. Tests were selected on the basis of each child’s estimated developmental age. To minimize error, a pre-determined protocol that specified the order of testing was followed. Results were reported as developmental age-equivalents to enable determination of the increase or decrease of skills over time.

### Statistical analysis

This is a retrospective and descriptive analysis of signs and symptoms of Hurler syndrome. For some data (developmental and growth) a cross sectional and longitudinal analysis was performed. Graphs were generated for each developmental domain by plotting the age-equivalent score (y-axis) against the patient’s actual age at the time of testing (x-axis). The developmental graphs also show a group mean curve, which was estimated using a linear polynomial model or a non-linear monomolecular model. The monomolecular model seemed more appropriate as it estimates three parameters: a starting place (fixed at 0), plateau, and rate at which the outcome reaches the plateau.

## Results

### Patient characteristics

Of the 55 patients with Hurler syndrome evaluated between 2000 and 2013, there were 23 boys (41.8%) and 32 girls (58.2%). Forty-four were evaluated once (*n* = 37) or twice (*n* = 7) at our clinic prior to transplantation. A medical record review was used to gather pre-transplantation information for the 11 patients who were not evaluated in our clinic prior to transplantation. One patient received bone marrow transplantation from a related donor, and 43 received umbilical cord blood transplantation. Five patients also received ERT prior to transplantation. The outcomes for a subset of these transplant patients have been reported previously [16, 17]. Only one patient has died due to natural progression of disease (age, 8.9 years). Genotypic data available for a subset of this cohort have been reported previously [16].

**Fig. 1:**
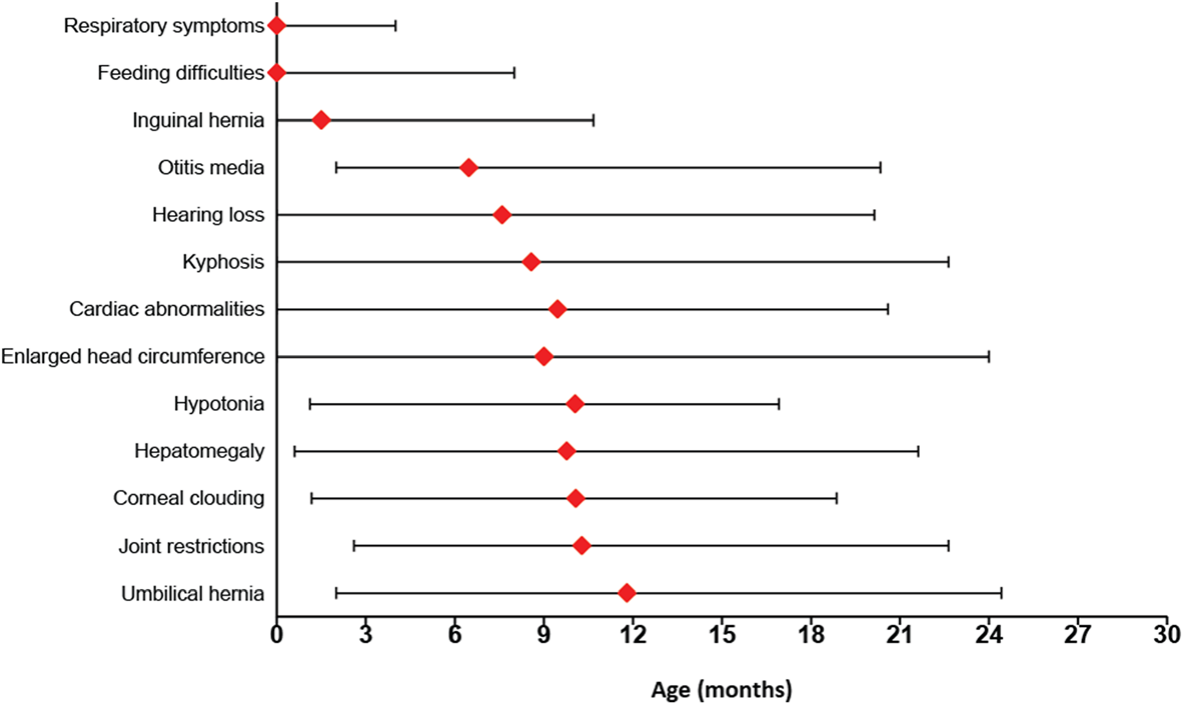
Median age of symptom onset with minimum to 9th decile range

**Fig. 2:**
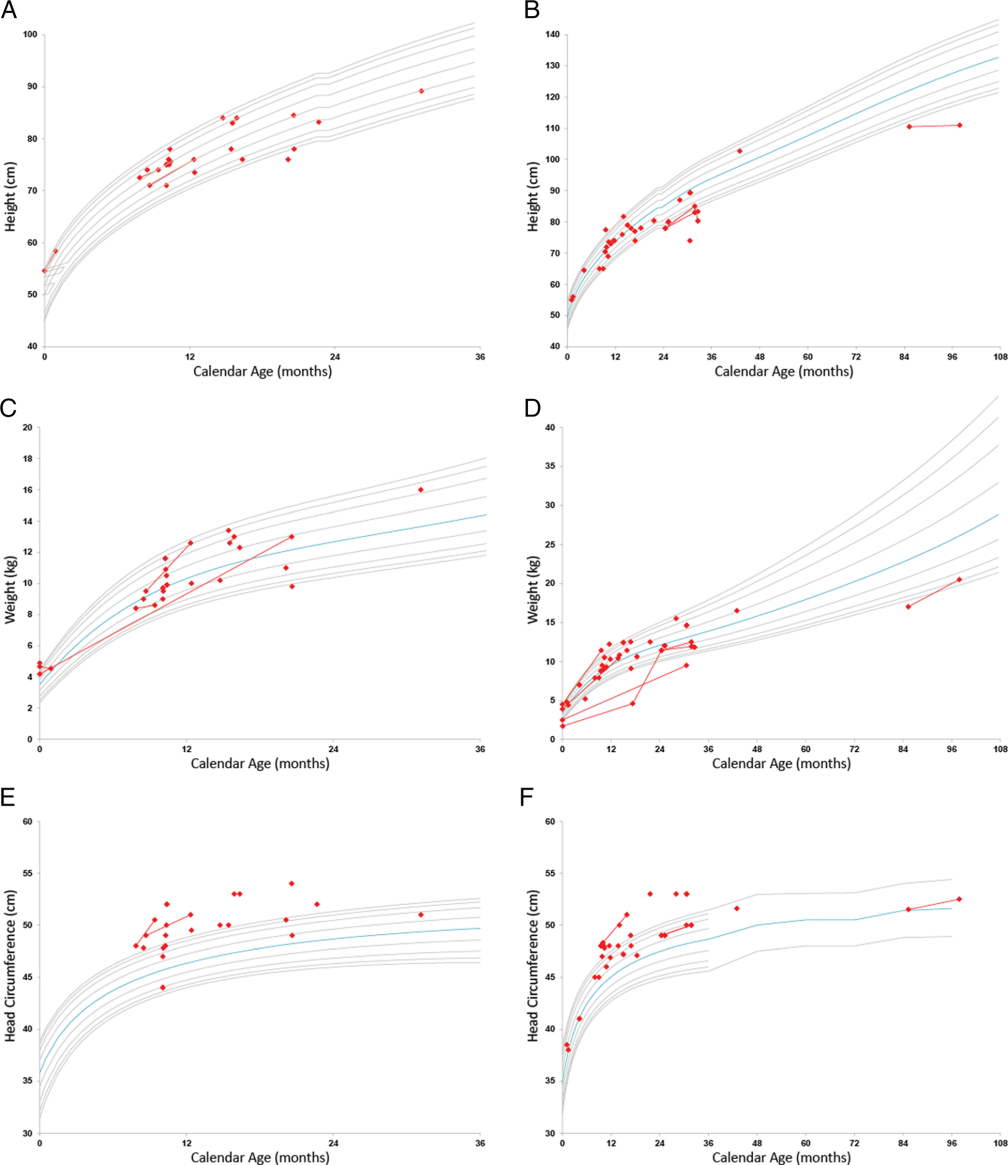
Growth charts for height, weight, and head circumference by gender. Gray lines show the population percentiles 3%, 5%, 10%, 25%, 75%, 90%, 95%, and 97%. The blue line represents the 50th percentile. Each patient is represented by either a dot (single evaluation) or line (longitudinal data). **a** Height-boys, **b** height-girls, **c** weight-boys, **d** weight-girls, **e** head circumference-boys and **f** head circumference-girls

Information about medical history, including the timing of symptom onset (Fig. 1), was available for all 55 patients. However, physical examination findings were available only for the 44 patients who had not undergone transplantation at the time of the initial evaluation at this specialized program (Table 1). Growth data for this cohort, including longitudinal measures of height, weight, and head circumference, are shown in Fig. 2.

**Table 1:** Medical history, surgical history, and physical examination findings

Medical and surgical history (*n* = 55)		Physical examination findings (*n* = 44)	
*Medical history*			
Acute or chronic otitis media	89.1%	Joint restrictions	93.2%
Noisy breathing or snoring	85.5%	Corneal clouding	81.8%
Hernia	63.6%	Kyphosis	77.3%
Respiratory infections	54.5%	Hypotonia	61.4%
Swallowing difficulty	45.5%	Hepatomegaly	61.4%
Apnea	32.7%	Transmitted upper respiratory sounds	56.8%
Reflux	30.9%	Nasal congestion	52.3%
Latching or sucking difficulty	21.8%	Protuberant or distended abdomen	45.5%
Diarrhea	18.2%	Nasal drainage	40.9%
Reactive airway disease	16.4%	Muscle weakness	29.5%
Constipation	12.7%	Gingival hypertrophy	22.7%
Laryngomalacia	9.1%	Murmur	22.7%
*Surgical history*		Abnormal palate	20.5%
Adenoidectomy	72.7%	Sacral dimple	18.2%
Tonsillectomy	61.8%	Mongolian spots	15.9%
Tympanostomy tubes	78.2%	Hirsutism	13.6%
Ventriculoperitoneal shunt	25.5%	Strabismus	9.1%

Information about medical and surgical history was available for all 55 patients. Physical examination findings were available for the subset of patients (*n* = 44) who were evaluated at our center prior to transplantation

### Initial presentation and diagnosis

Data from the prenatal period (available for 41/55 patients) were unremarkable in most cases. Eight of the 55 patients (14.5%) were born before 37 weeks of gestation, and 24 (43.6%) were delivered via cesarean section. Seven patients (12.7%) were noted to have orthopedic abnormalities at birth including kyphosis (*n* = 4), talipes equinovarus (*n* = 2), sacral dimple (*n* = 1), pectus carinatum (*n* = 1), and pectus excavatum (*n* = 1). Other congenital anomalies included hernias (*n* = 5), cardiac defects (*n* = 3), and hypospadia (*n* = 2). Fifteen infants (27.3%; 3 premature, 12 full-term) had hyperbilirubinemia during the neonatal period.

Most patients developed signs of disease during the first months of life. The proportion of infants with at least one of the following symptoms was 83.6% (*n* = 46) by 1 month and 98.2% (*n* = 54) by 6 months: respiratory difficulties, otitis media, hearing loss, corneal clouding, hernias, organomegaly, hypotonia, feeding difficulties, joint restrictions, enlarged head circumference, cardiac disease, and kyphosis. Nonetheless, diagnostic delays were common, and the patients in this cohort were not diagnosed with MPS I until a median of 9.3 months. The most common symptoms that prompted the workup leading to diagnosis were kyphosis (*n* = 19; 34.5%), delayed acquisition of motor or language milestones (*n* = 17; 30.9%), enlarged head circumference (*n* = 15; 27.3%), corneal clouding (*n* = 10; 18.2%), and dysmorphic facial features (*n* = 9; 16.4%).

### Sensory functioning

#### Audiological

Hearing loss and otological disease were among the earliest disease manifestations in this cohort. Fifteen of the 33 patients with available results (45.5%) failed their hearing screen at birth. Audiological evaluations, which most commonly included behavioral audiometry (*n* = 23) and/or auditory brainstem response testing (*n* = 19), were subsequently performed in 42 patients. Hearing loss was evident in 32 patients (76.2%), whereas 5 patients (11.9%) had normal hearing, and results were inconclusive for the remaining 5 patients. Of the 20 patients with available information about type of hearing loss, 11 (55.0%) had predominantly sensorineural deficits, and the other patients had predominantly conductive (*n* = 7; 35.0%) or mixed (*n* = 2; 10.0%) hearing loss. Median age at the first failed hearing examination was 7.6 months. Symptoms of middle ear pathology were present in the vast majority of patients. Forty-nine patients (89.1%) had a history of chronic middle ear effusion and/or acute otitis media, which began at a median of 6.5 months. These symptoms led to tympanostomy tube insertion for 43 patients (78.2%) at a median age of 10.9 months.

#### Ophthalmologic

Corneal clouding was the most frequent ophthalmological finding (*n* = 36; 81.8%) and was present in all patients older than 15 months at the time of evaluation. Corneal clouding was diagnosed at a median age of 10.1 months and was frequently preceded by a history of photophobia. Pupillary responses were normal in all patients who were evaluated. Four patients had strabismus.

### Feeding and oral-motor manifestations

Feeding difficulties affected 39 patients (70.9%) in this cohort. Twelve patients (21.8%) had problems with latching on to the breast or sucking during the first month of life, and 17 (30.9%) developed symptoms of gastroesophageal reflux. Swallowing issues were present to varying degrees in 25 patients (45.5%) and commonly manifested as episodes of gagging or choking on food (*n* = 19) and/or aspirating or coughing with liquids (*n* = 8). Seven children (12.7%) were also noted to have strong aversions to textured foods. Physical characteristics that may have contributed to feeding difficulties included macroglossia (*n* = 13; 29.5%), and palate abnormalities (*n* = 9; 20.5%). Ten patients (22.7%) had gingival hypertrophy. A number of patients also had problems with bowel movements such as diarrhea (*n* = 10; 18.2%) and/or constipation (*n* = 7; 12.7%).

### Somatic manifestations

#### Respiratory

Respiratory difficulties typically began soon after birth. Fourteen infants (25.5%; 6 premature, 8 full-term) required respiratory support during the neonatal period. An additional 18 patients (32.7%) developed other respiratory symptoms during the first month of life, including signs of upper airway obstruction (*n* = 16) and infections (*n* = 2).

Even among patients with an unremarkable neonatal course, the vast majority ultimately developed respiratory symptoms. Forty-seven patients (85.5%) exhibited noisy breathing or snoring, and 18 (32.7%) had apneic episodes. Laryngomalacia was diagnosed in five patients, one of whom required supraglottoplasty at 4 months of age. Concerns about airway obstruction frequently resulted in adenoidectomy (*n* = 40; 72.7%; median age, 12.3 months) and/or tonsillectomy (*n* = 34; 61.8%; median age, 14.5 months). Nine patients (16.4%) also required treatment with nebulizers to address signs of comorbid reactive airway disease.

Prior to transplantation 30 patients (54.5%) developed respiratory infections, including pneumonia (*n* = 8; 14.5%). Many patients experienced persistent upper respiratory symptoms even after the cessation of active infections. On examination, nasal congestion (*n* = 23; 52.3%), drainage (*n* = 18; 40.9%), and transmitted upper respiratory sounds (*n* = 25; 56.8%) were frequently noted (Table 1).

### Cardiovascular

Of the 42 patients with available echocardiogram and/or electrocardiogram results, abnormalities were detected in 35 (83.3%). Twenty-six patients (61.9%) had valve abnormalities, diagnosed at a median age of 13.0 months. Valve insufficiency was present in 21 patients (50.0%). The mitral valve was most commonly affected (*n* = 19), followed by the tricuspid (*n* = 7) and aortic (*n* = 3) valves. Fifteen patients (35.7%) had thickened or dysplastic valves. Ten patients (23.8%) had ventricular hypertrophy. Other findings included patent foramen ovale or atrial septal defects (*n* = 9), ventricular dilation (*n* = 4), patent ductus arteriosus (*n* = 4), supraventricular tachycardia (*n* = 4), atrial dilation (*n* = 2), pericardial effusion (*n* = 2), and ventricular septal defects (*n* = 1). At the time of evaluation 10 of 44 patients (22.7%) had audible murmurs. The median age at the initial detection of any cardiac abnormality was 9.5 months.

### Other somatic manifestations

More than three-fourths of patients with available data were found to have kyphosis on examination (*n* = 34; 77.3%; Table 1), which was identified at a median age of 8.6 months. Hepatomegaly, which was present in 27 patients (61.4%), was detected slightly later, at a median of 9.8 months. Twenty patients (45.5%) were noted to have a protuberant or distended abdomen. Most patients (*n* = 35; 63.6%) also developed hernias. Inguinal hernias were typically detected soon after birth, at a median of 1.5 months (*n* = 20; 36.4%), whereas umbilical hernias did not appear until a median of 11.8 months (*n* = 26; 47.3%). Other findings included sacral dimples (*n* = 8), Mongolian spots (*n* = 7), hirsutism (*n* = 6), hemangiomas (*n* = 3), and café au lait spots (*n* = 1).

### Neuromuscular manifestations

Most patients presented with neuromuscular abnormalities. On examination, 27 (61.4%) were found to have hypotonia, which was initially detected at a median age of 10.1 months. Weakness of the trunk and/or extremities was noted in 29.5% (*n* = 13). The vast majority of patients (*n* = 41; 93.2%) had restrictions in one or more joints, most commonly in the shoulders. Joint restrictions were initially noted at a median of 10.3 months. Upper extremity deep tendon reflexes, assessed in 39 patients, were diminished (1+) in 11 (28.2%), normal (2+) in 27 (69.2%), and brisk (3+) in 1 (2.6%). Lower extremity deep tendon reflexes, assessed in 41 patients, were diminished in 7 (17.1%), normal in 27 (65.9%), and brisk in 6 (14.6%). One patient had clonus in the lower extremities.

### Head growth and neuroimaging findings

At the time of evaluation, head circumference was above the 50th percentile for most children and above the 95th for many (Fig. 2). Concerns about enlarged head size emerged at a median of 9.0 months. Fourteen children (25.5%) required placement of a ventriculoperitoneal shunt for hydrocephalus at a median of 10.7 months.

Pre-transplant brain MRI scans were available for 37 patients. The most frequent findings were white matter abnormalities (*n* = 21; 56.8%), dilated Virchow-Robin perivascular spaces (*n* = 22, 59.5%), volume loss (*n* = 17, 45.9%), dilated ventricles (*n* = 15, 40.5%), pituitary sella abnormalities (*n* = 5, 13.5%), and enlarged extra-axial cerebrospinal fluid spaces (*n* = 6, 16.2%). White matter changes were noted as early as 6.8 months of age.

### Neurodevelopmental functioning

**Fig. 3:**
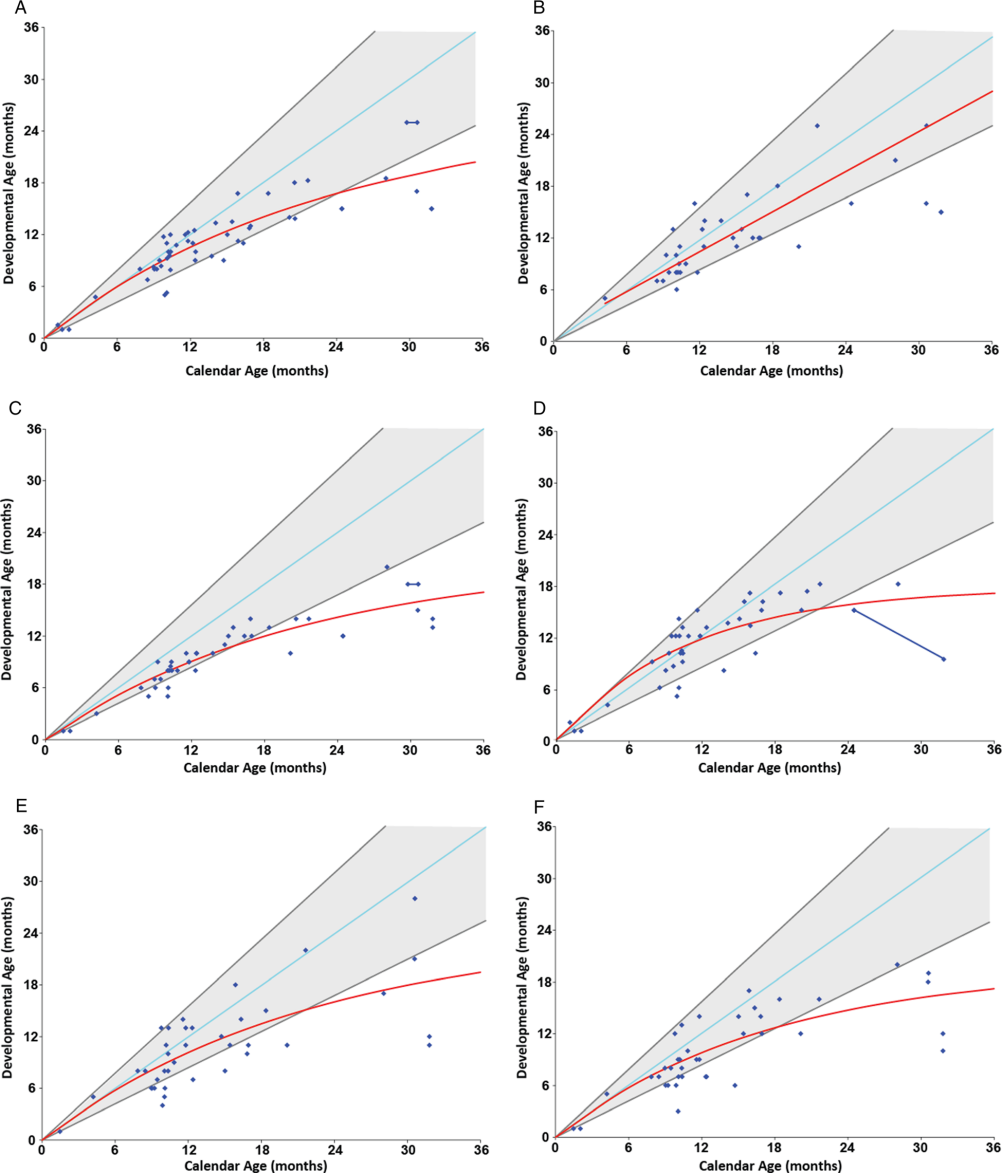
Developmental charts. The figures plot individual age-equivalent scores (y-axis) against the actual age of the patient (x-axis) for each of the developmental domains. The red line represents the estimated group mean. **a** Cognitive development, **b** adaptive behavior, **c** gross motor, **d** fine motor, **e** receptive language and **f** expressive language

The results of developmental testing are shown in Fig. 3. Developmental age was plotted against chronological age for each patient evaluated.

#### Cognition

Cognitive function was preserved in most children evaluated during the first year of life. Within this age group, only three were outside the normal range in measures of cognition. However, as a group these patients showed lower cognitive functioning than normally developing children. Cognitive development in children with Hurler syndrome started to deviate from typical development (gray lines) at approximately 9 months of age (Fig. 3a).

#### Adaptive behavior

Adaptive behavior was normal in most children evaluated during the first year of life (Fig. 3b). However, as a group they tested below the normal 50th percentile, and this area of development become more abnormal over time. Adaptive behavior scores tended to be superior to cognitive and gross motor scores.

#### Motor

Gross motor function was the weakest area of development in this cohort. Deficits generally became apparent during the first year of life (Fig. 3c). In addition, many patients failed to acquire early gross motor milestones on time [18]. According to parent reports, 46.5% (20/43) of the children did not sit independently until at least 8 months of age, 30.0% (6/20) did not crawl until at least 12 months of age, and 40.0% (8/20) did not walk independently until at least 16 months of age.

Fine motor skills were superior to gross motor skills in most children (Fig. 3d). Within the first year of life there was no obvious difference between the group mean and typical development. However, starting at 12 months of age the group mean (red line) began to plateau, becoming more abnormal after 24 months.

#### Language

Delays in both receptive and expressive language often appeared during the first year of life (Fig. 3e & f). Seven (33%) of the 21 children evaluated between 6 and 12 months of age showed below normal language scores in receptive and expressive language.

## Discussion

Information about the natural history of Hurler syndrome may help clinicians to manage infants diagnosed with MPS I through newborn screening. With this study, we characterized the early onset and progression of neurological and somatic disease in patients with Hurler syndrome. This represents, to our knowledge, the largest and most comprehensive study of the natural progression of early symptoms of Hurler syndrome based on systematic physical and neurodevelopmental evaluations performed at a single center.

The patients in this cohort were diagnosed with MPS I at a median age of nine months. Our group has previously demonstrated that transplantation of patients with Hurler syndrome during the first nine months of life is associated with normal cognitive development, while those that are transplanted later show long-term deficits [6]. In light of our finding that 98% of these patients showed signs of disease during the first six months of life, this represents an unacceptable delay in diagnosis. Although the retrospective nature of this study precluded the determination of causal relationships, the delay in treatment likely affected neurodevelopmental outcomes in these patients. Recurrent otitis media, hearing loss, and hypertrophy of the adenoids, tonsils, and tongue may have contributed to expressive and receptive language delays. Similarly, the presence of neuromuscular symptoms such as joint restrictions and hypotonia likely hindered gross motor function, which was the weakest area of development in these patients.

Although the addition of MPS I to the Recommended Uniform Screening Panel constitutes an important step towards minimizing diagnostic delays, the implementation of widespread newborn screening for this disorder may take years, and many patients will likely continue to be diagnosed with MPS I on the basis of clinical presentation. Thus, increased awareness among primary care providers about the initial symptoms of this disorder is essential to facilitate early identification and treatment of affected patients. More than a quarter of this cohort failed the newborn hearing screen, and several had congenital anomalies such as cardiac defects, orthopedic deformities, and inguinal hernias. The vast majority of patients also developed respiratory, otological, and/or feeding difficulties during the first six months of life. The nonspecific nature of these early symptoms likely contributed to diagnostic delays; otitis media, which occurred in nearly 90% of this cohort beginning a median of 6.5 months, has been found to affect more than a third of all newborns during the first six months of life [19]. Likewise, feeding difficulties, which were present in more than 70% of this cohort, have been reported to affect 25% of the typically developing population [20]. Pediatricians should be made aware that the occurrence of these nonspecific symptoms in conjunction with other characteristic signs of MPS I – such as kyphosis, corneal clouding, and hepatomegaly, which had median onset ages of 8–10 months in this cohort – warrants immediate evaluation for a lysosomal storage disorder.

The implementation of NBS for this disorder shows promise to minimize diagnostic delays. Nonetheless, the clinical management of screen-positive infants will likely pose a number of challenges. The high prevalence of early symptoms in this cohort suggests that infants diagnosed with MPS I through NBS should be monitored closely for signs and symptoms of disease onset, since early hematopoietic stem cell transplantation improves neurodevelopmental outcomes [6]. Given the high incidence of feeding difficulties, upper respiratory symptoms and hearing problems in this cohort, we recommend that when all of these are identified in the first 6 months of life in an infant who screens positive for MPS I, the patient should be referred for further testing to a specialized center to evaluate the presence of neurological disease and make recommendations about the need for transplantation. Although routine genetic and biochemical tests are, individually, limited in their ability to predict MPS I phenotype, approaches that integrate clinical and laboratory data may have greater prognostic value. Kingma et al. (2013) proposed a three-part algorithm that combined the results of genetic testing, residual enzyme activity levels, and information about two symptoms (upper airway obstruction and inguinal hernias) during the first month of life to predict whether an infant with MPS I will develop the Hurler phenotype [12]. We propose adding to this algorithm otitis media, sensorineural hearing loss and feeding difficulties, since they commonly appeared soon after birth and could potentially serve as early markers of disease severity in patients with Hurler syndrome. However, to validate these and any other potential prognostic clinical markers, the progression and timing of symptom onset will need to be compared between patients with Hurler syndrome and those with attenuated forms of MPS I. Unfortunately, the few studies that have described the natural history of attenuated MPS I phenotypes did not focus on disease manifestations during the first year of life [1, 21, 22] Thus, further research is needed in this area.

As a retrospective review of clinical data, this study is subject to a number of limitations. Complete medical records were not available for all patients. Moreover, information about several symptoms such as feeding difficulties, ear infections, and noisy breathing was obtained in part through parent interviews, which are subject to recall bias. For a number of other disease manifestations, the timing of onset was ascertained by reviewing available medical records. Since delays may occur between the emergence of a symptom and its detection by a medical professional, these symptoms may have appeared sooner than our estimates suggest, further highlighting the need for early and vigilant monitoring of all infants with MPS I. The implementation of newborn screening for this disorder may enable prospective studies of the timing of symptom onset in affected infants, thus overcoming many of the limitations of the present study. Despite the study’s limitations, the use of a predetermined protocol for physical examinations and developmental testing carried out by professionals experienced in working with children with neurodegenerative disorders represents a major strength of the present study.

## Conclusion

This study characterized the early progression and timing of symptom onset in infants with Hurler syndrome. Information about the clinical course of this disorder is needed to aid in the management of affected patients and to facilitate assessment of treatment effectiveness, especially in the context of NBS. It is hoped that a better understanding of the early progression of Hurler syndrome will result in improved outcomes for affected children.

## Abbreviations

CNS: Severe central nervous system
ERT: Enzyme replacement therapy
HSCT: Hematopoietic stem cell transplantation
MPS I: Mucopolysaccharidosis type I
MRI: Magnetic resonance imaging
NBS: Newborn screening
